# The protocol for the Be Our Ally Beat Smoking (BOABS) study, a randomised controlled trial of an intensive smoking cessation intervention in a remote Aboriginal Australian health care setting

**DOI:** 10.1186/1471-2458-12-232

**Published:** 2012-03-23

**Authors:** Julia V Marley, David Atkinson, Carmel Nelson, Tracey Kitaura, Dennis Gray, Sue Metcalf, Richard Murray, Graeme P Maguire

**Affiliations:** 1 The Rural Clinical School of Western Australia, The University of Western Australia, Cnr Anne & Dora Street, PO Box 1377, Broome, WA, 6725, Australia; 2 Kimberley Aboriginal Medical Services Council, Cnr Anne & Dora Street, PO Box 1377, Broome, WA 6725, Australia; 3 Derby Aboriginal Health Service, 1 Stanley Street, PO Box 1155, Derby, WA 6728, Australia; 4 National Drug Institute, Curtin University, GPO Box U1987, Perth, WA 6845, Australia; 5 School of Medicine and Dentistry, James Cook University, Cairns, Queensland 4870, Australia; 6 Baker IDI, Alice Springs, Northern Territory 0871, Australia

**Keywords:** Indigenous, Aboriginal, Torres Strait Islander, Randomised controlled trial, Smoking cessation, Study protocol, Be Our Ally Beat Smoking (BOABS) Study

## Abstract

**Background:**

Australian Aboriginal peoples and Torres Strait Islanders (Indigenous Australians) smoke at much higher rates than non-Indigenous people and smoking is an important contributor to increased disease, hospital admissions and deaths in Indigenous Australian populations. Smoking cessation programs in Australia have not had the same impact on Indigenous smokers as on non-Indigenous smokers. This paper describes the protocol for a study that aims to test the efficacy of a locally-tailored, intensive, multidimensional smoking cessation program.

**Methods/Design:**

This study is a parallel, randomised, controlled trial. Participants are Aboriginal and Torres Strait Islander smokers aged 16 years and over, who are randomly allocated to a 'control' or 'intervention' group in a 2:1 ratio. Those assigned to the 'intervention' group receive smoking cessation counselling at face-to-face visits, weekly for the first four weeks, monthly to six months and two monthly to 12 months. They are also encouraged to attend a monthly smoking cessation support group. The 'control' group receive 'usual care' (i.e. they do not receive the smoking cessation program). Aboriginal researchers deliver the intervention, the goal of which is to help Aboriginal peoples and Torres Strait Islanders quit smoking. Data collection occurs at baseline (when they enrol) and at six and 12 months after enrolling. The primary outcome is self-reported smoking cessation with urinary cotinine confirmation at 12 months.

**Discussion:**

Stopping smoking has been described as the single most important individual change Aboriginal and Torres Strait Islander smokers could make to improve their health. Smoking cessation programs are a major priority in Aboriginal and Torres Strait Islander health and evidence for effective approaches is essential for policy development and resourcing. A range of strategies have been used to encourage Aboriginal peoples and Torres Strait Islanders to quit smoking however there have been few good quality studies that show what approaches work best. More evidence of strategies that could work more widely in Indigenous primary health care settings is needed if effective policy is to be developed and implemented. Our project will make an important contribution in this area.

**Trial Registration:**

Australian New Zealand Clinical Trials Registry (ACTRN12608000604303)

## Background

While Australian Aboriginal peoples and Torres Strait Islanders (Indigenous Australians) historically had access to nicotine containing plants growing naturally, heavy use was presumably rare. However the addictive nature of nicotine was readily exploited by Europeans [1], with tobacco being supplied as part of rations on cattle stations and missions until the 1960s [2]. Tobacco smoking is now normalised in Indigenous Australian society [1]. In 2007, the prevalence of smoking in the non-Indigenous population was 19% [3]. In the 2004 National Aboriginal and Torres Strait Islander Health Survey, 52% of Indigenous Australians reported being smokers [4]. In contrast, the 2007 National Drug Strategy Household Survey reported that 34% of Indigenous Australians were smokers [3] whereas the current prevalence is estimated to be in excess of 45% [5]. Over 35% of Indigenous Australian people aged between 12 and 17 report regular smoking and smoking prevalence increases with age [6,7]. This early regular tobacco consumption can lead to increased dependence upon nicotine and reduces the success of cessation attempts later in life [8]. A number of reports indicate that Indigenous Australians are less likely to stop smoking compared with non-Indigenous people [9].

Smoking among Aboriginal peoples and Torres Strait Islanders contributes significantly to higher rates of hospitalisation and death from tobacco-related conditions and poorer self-reported health [10-12]. Much of the health disparity between Indigenous and non-Indigenous people in Australian is attributable to conditions that are either due to or made worse by tobacco consumption. Smoking substantially increases the risk of both macrovascular and microvascular complications of diabetes and may have a role in the development of type 2 diabetes [10]. Indigenous health professionals are reported to be more likely to smoke than non-Indigenous health professionals and to have lower rates of awareness about the detrimental effects of smoking behaviour [13].

Helping established tobacco smokers to stop smoking and maintain this is notoriously difficult in any setting. Interventions to help smokers to quit have, even in the controlled setting of experimental randomised controlled trials (RCTs), a success rate of at most 25% at 12 months and are more typically substantially lower. The quit rates in primary health care settings, particularly when health workers approach clients to quit, are likely to be lower than in settings where clients have specifically sought help [2]. Motivation to quit prior to being involved in an intervention did not predict success in quitting [14,15]. Several RCTs have demonstrated that 5-22% of participants who were not interested in quitting at the start of their participation had quit smoking at the follow-up (reviewed in [16]). The data from these studies suggest that quitting can occur among those who appear to be relatively unmotivated smokers and can be encouraged by intervention and the use of pharmacotherapy (e.g. nicotine replacement therapy (NRT)).

Helping Aboriginal peoples and Torres Strait Islanders to quit smoking is complex, due in part to the multiple life stressors experienced by them [17], and the need to identify and support local champions [18]. Integrated services provided by Aboriginal Community Controlled Health Services (ACCHS), which provide a 'culturally safe' environment have been shown to be successful, and interventions can reduce risk [19].

Given the stark and persisting health inequalities borne by Indigenous Australians [20] - despite existing evidence-based interventions - it is imperative research is undertaken in this setting to provide insights into how best to respond to primary and secondary disease prevention. RCTs provide high-quality research and evidence, and can be undertaken in this setting. In 2002 Kimberley ACCHS took part in a double blind, multi-centre, RCT that was conducted by the National Aboriginal Community Controlled Health Organisation (NACCHO Ear Health Trial) [21]. The research methodology of this RCT placed Aboriginal people in control of their own research [22]. RCTs when carried out in ACCHS need to be simple, modified to the local setting, and embedded in primary health care for them to be successful and sustainable.

This study aims to test the efficacy of a culturally appropriate multidimensional intensive smoking cessation intervention provided by Aboriginal researchers in helping Aboriginal peoples and Torres Strait Islanders to become non-smokers.

### Hypothesis

A culturally appropriate, multidimensional, intensive smoking cessation intervention, provided by trained Aboriginal researchers, will be more effective than current standard practice in achieving and sustaining cessation to tobacco consumption among Aboriginal peoples and Torres Strait Islanders.

## Methods/Design

### Objectives

The primary objectives of this study are to 1) develop a locally-tailored, intensive, multidimensional smoking cessation program utilising identified Aboriginal smoking prevention officers, and 2) to determine the effectiveness of this program through implementation of a RCT comparing it with a standard primary care based brief intervention strategy in two Kimberley ACCHS. Secondary objectives include encouraging research interest and capacity among Aboriginal health staff and to build skills in Good Clinical Practice as they relate to the conduct and evaluation of a RCT. The Aboriginal researchers employed on the study named it the BOABS (Be Our Ally, Beat Smoking) Study.

### Study design

**Figure 1:**
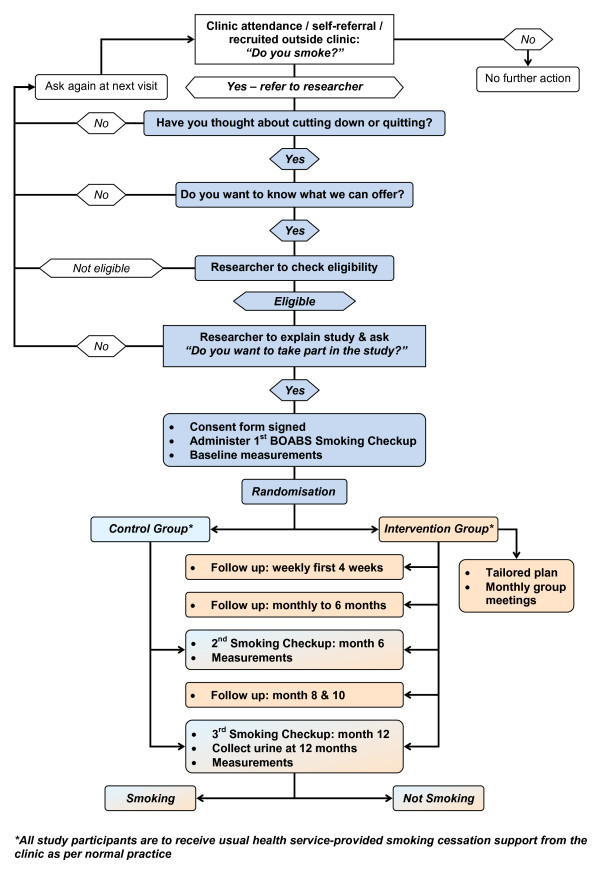
**Flowchart for a randomised controlled trial of a culturally appropriate, multidimensional, intensive smoking cessation intervention provided by trained Aboriginal researchers**.

This study is a parallel, randomised controlled study. Participants are randomly allocated to one of two study arms: the 'intervention' group who participates in the BOABS program over 12 months, or the control group who are allocated to 'usual care' (i.e. they do not participate in the BOABS program). Aboriginal researchers are trained to deliver the BOABS program, the goal of which is to help Aboriginal people quit smoking. Data collection occurs at baseline (when participants enrol in the study), and at six and 12 months following enrolment. The primary outcome of interest is the proportion of subjects who self-report smoking cessation with urinary cotinine validation at 12 months. Figure 1 outlines the flow of participants through the study.

### Study populations

**Figure 2:**
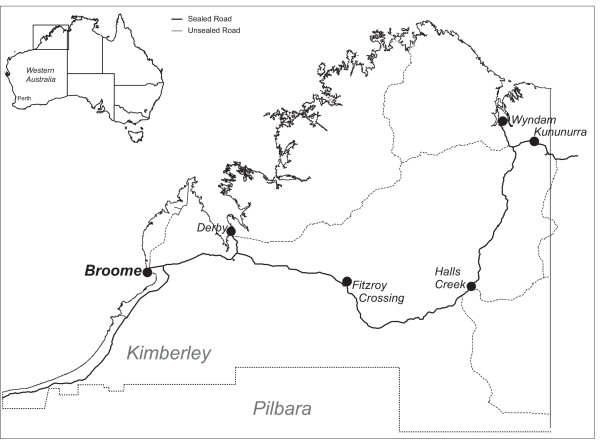
**Sites where the Be Our Ally Beat Smoking (BOABS) Study is being conducted, the Kimberley, Western Australia**.

This study is being conducted in Derby and Kununurra in the remote Kimberley region of far north Western Australia (see Figure 2). The Kimberley is one of the most isolated and sparsely populated areas of Australia with a resident population of 29,298 [23] spread over 423,517 km^2^. While Indigenous Australian people are socioeconomically disadvantaged everywhere in Australia [24], remote Indigenous people, including most residents of the Kimberley and the adjacent Northern Territory (NT), are in the lowest socioeconomic quartile of Indigenous people [24,25], the most disadvantaged group within the most disadvantaged population in Australia. The target population for this study are Aboriginal and Torres Strait Islander smokers who resided in or near these two towns. The sampling unit is Aboriginal and Torres Strait Islander smokers (aged 16 years and over).

The Kimberley region extends 1100 km from east to west and 850 km from north to south. The study sites are Derby Aboriginal Health Service in Derby and Ord Valley Aboriginal Health Service in Kununurra. The centralised coordinating site is the Kimberley Aboriginal Medical Services Council in Broome

### Inclusion criteria

Participation in the trial is based on the following inclusion criteria:

• Aboriginal and/or Torres Strait Islander

• ≥ 16 years of age

• Self-reported current smoker or quit within 2 weeks of recruitment

• Thinking about cutting down or quitting smoking

• A regular client of Derby Aboriginal Health Service (DAHS) in Derby or Ord Valley Aboriginal Health Service (OVAHS) in Kununurra

### Exclusion criteria

People are excluded from participation in the trial based on the following criteria:

• Unable to provide informed consent

• A health condition that would prevent them from completing the trial

• Unlikely to be available for follow up at 12 months

### Randomisation: allocation, concealment and sequence generation

Block randomisation (36 participants per block × 6 blocks) is used to randomly assign participants to Group 1 (control group) or Group 2 (intervention group). Each site has 216 envelopes allocated. Each block has 24 participants randomly assigned to Group 1 and 12 to Group 2 using a computer generated random list of dichotomous outcomes (control or intervention) generated for each site using Excel 2007 software (Microsoft, Redmond, WA, USA). While only 5 blocks are required an extra block is generated to allow for contingencies that might require extra participants to be recruited. Envelopes are filled and sealed by an individual who is not involved with the study or its analysis. Envelopes are kept off-site and under the supervision of administrative staff based at a centralised coordinating site (the Kimberley Aboriginal Medical Services Council in Broome). Allocation occurs via telephone with envelopes being opened in sequential order for each site only once a participant is consented and enrolled in the study, eligibility is confirmed, an identification number is, assigned and the first questionnaire completed.

### Blinding

Due to the nature of the intervention and the 2:1 weighting of participants in the control and intervention arms, the participants, research team and local healthcare staff know whether each participant belongs to the control or intervention group. The staff performing the urinary cotinine assay are unaware of the allocation.

### Proposed intervention

Participants are told that they will be randomly assigned to a group that receives 'usual care', or a group that receives extra support visits by an Aboriginal smoking cessation officer, and that a questionnaire will be administered when they enrol, and at six and 12 months following enrolment.

#### Participants assigned to the control group

Participants allocated to the control group receive the usual practice of the service, including but not limited to advice from clinical staff regarding the harmful effects of smoking, advice regarding quitting, pharmacotherpay, self-initiated follow up, and access to usual services offered by the service (e.g. at DAHS clients are offered participation in lifestyle and primary prevention education classes).

#### Participants assigned to intervention group

Participants allocated to the intervention group also receive the usual health service-provided smoking cessation support, as above. In addition to this they are provided with smoking cessation counselling at face-to-face visits, which will be scheduled weekly for the first four weeks, monthly to 6 months and two monthly to 12 months. During the intervention the Aboriginal researchers are to concentrate on smoking cessation. Face-to-face follows up can take place at the clinic or other location suitable to the participant (e.g. home, work). While face-to-face contact is the default option, this may have to be adjusted if a participant's circumstances makes this difficult (e.g. a full-time worker may be offered phone contact for some of the follow up visits). If a participant does not attend for a follow up visit, the Aboriginal researcher is to initiate active follow up within 48 hours through phone or home visit to determine the reason for non-attendance and where possible determine solutions to encourage ongoing participation.

Participants in the intervention group are also encouraged to participate in a monthly smoking cessation support group, which include: 1) additional smoking cessation support and advice including cognitive and behavioural approaches to 'quitting'; 2) opportunities for information sharing/sharing of barriers and solutions; 3) a light healthy meal and 4) information sharing and advice on other aspects of health and well-being including management and prevention of common chronic diseases, which will be delivered by appropriately trained people. The group therapy occurs at both sites. This is a rolling group, with participants continually entering and leaving the group.

Content to be delivered by Aboriginal researchers:

• Individual

○ Motivational interviewing (e.g. what do you like/do not like about smoking)

○ Triggers for smoking/diversions & strategies to deal with them

○ Action plans - preventing and dealing with short term relapses (termed 'slipups')

○ Discussion regarding the positives of smoking cessation (e.g. saving money)

○ Pharmacotherapy (e.g. NRT)

○ Identification of factors driving smoking and case management to link participants to additional non-health service, community-based support agencies (e.g. alcohol counselling)

○ Smoking cessation-associated weight gain - generic discussions regarding potential weight gain and providing education and advice (e.g. 'healthy' eating, exercise)

• With the health care clinic medical officer/general practitioner

○ Organise weekly case conference to review all active participants in the intervention group

• Monthly group meeting

○ Researcher conducted

○ Including dietician input for associated weight control advice

• Service mapping and linkages

○ Identify and develop referral networks to help address consequences of tobacco cessation and drivers of tobacco consumption (e.g. alcohol, domestic violence, stress, mental illness, weight changes, etc.)

### Outcome measures

Primary endpoint:

• The proportion of participants who were not smoking tobacco at 12 months as determined by self-report and urinary cotinine level consistent with no recent tobacco consumption [26].

Secondary endpoints:

• The proportion of participants who were not smoking at 6 months as determined by self-report.

• The proportion of participants reporting excellent or very good health using a self-reported health questionnaire [27].

• The proportion of participants who reported 20% or greater reduction in the number of cigarettes smoked each week.

• Process evaluation indicators: a mix of quantitative and qualitative measures to assess how well the intervention program was implemented according to the protocol e.g. number of 'face-to-face' meetings completed, challenges and solutions in delivering the program.

### Sample size

The sample size estimation is based on a two-sided alpha of 0.05, beta of 0.2 (power 0.8), ratio of intervention to control participants of 1:2, and efficacy of maintaining smoking cessation at 12 months of 3% in the control group and 13% in the intervention group. Based on these assumptions the sample size required will be 106 in the intervention group and 212 in the control group across both sites. Using a conservative estimate of 50% of the clients being smokers, we will be recruiting less than 20% of regular DAHS and OVAHS clients for the trial. To take into account those lost to follow up (e.g. deaths), the aimed sample size is 120 in the intervention group and 240 in the control group across both sites.

### Recruitment strategies

Participants are being actively and opportunistically recruited. Active recruitment is facilitated by the Project Manager or Aboriginal researchers through incidental encounters in the community, family and community links. The participants do not need to see health care staff prior to being recruited, but are referred to the primary health care clinic afterwards.

Participants are being passively recruited through OVAHS and DAHS routine clinic visits as well as through running dedicated Adult Health Check clinics ('Aboriginal and Torres Strait Islander Adult Well Persons Check') for overdue clients - clients known or found to be current smokers are offered participation in the trial by clinic health workers: ***"Have you thought about cutting down or quitting? Do you want to know what we can offer?" ***(see Figure 1 for a flow diagram explaining the steps involved).

### Planned recruitment rate and risk of loss to follow up

We aim to recruit 12 people (approximately 8 control, 4 intervention) monthly for 15 months. This regular staggered recruitment is to ensure that a maximum of 38 participants will be followed up by the four half-time Aboriginal researchers in any one week period. This will allow for variations to the scheduled recruitment without placing too much pressure on resources. The trial should be completed in 26 months.

As it proved difficult for participants to complete the original six and 12 month questionnaires, an alternative, minimal data collection tool was created. If participants leave Derby or Kununurra the Aboriginal researchers attempt to administer the final questionnaire by alternate means (e.g. phone, Facebook) and arrange for a local health care clinic to collect urine samples.

If recruitment of 360 participants (120 intervention and 240 control) is proving difficult within the time frame of the trial, an interim analysis of primary endpoints will be performed. It should be noted that the requirement for an interim analysis will be at the discretion of the investigators and anticipated speed of recruitment. If required, an interim analysis is to be undertaken by the Data Safety and Monitoring Committee (DSMC) once 50% of the participants (60 in the intervention and 120 in the control group) have completed the study. If this demonstrates a difference that is deemed by the DSMC to be statistically and clinically significant then the DSMC will provide advice to the supervising Human Research Ethics Committees (HREC) regarding whether the trial should be terminated.

### Withdrawal criteria

Participants may be withdrawn from the study if one or more of the following occurs:

• Voluntary withdrawal: a participant can voluntarily withdraw at any time without having to provide a reason for doing so

• Death

• Significant illness requiring prolonged hospitalisation

• A serious and irreconcilable protocol violation (as determined by the investigators)

### Data management

All information is initially recorded by Aboriginal researchers on paper forms. Paper records are stored under numerical code in a locked filing cabinet only accessible to study personnel. All information collected from participants are treated as strictly confidential. Administrative staff will then transpose this information to a password-protected Access 2007 database (Microsoft, Redmond, WA, USA). De-identified data will be stored in password protected computers.

### Data monitoring

The DSMC is an ad hoc committee to be convened in the event of an expected adverse event, serious adverse event or protocol deviation. Any change or alteration from the procedures stated in the study protocol, consent document, recruitment process, or study materials (e.g. questionnaires) originally approved by the HREC are considered to be protocol deviations. Notification to the DSMC and reporting of the DSMC to the supervising HREC will occur if there is 1) any unexpected medical event in a participant involved in the trial as per the streamlined reporting scheme recommended by the National Health and Medical Research Council (NHMRC) and Australian Health Ethics Committee [28] or 2) a protocol deviation.

All recorded information will be assessed at least monthly by the Project Manager. The data stored on the relational database described above will be randomly assessed to make sure there are no errors in transcribing.

### Data analyses

Data from the trial will be entered into the database described above and then extracted into Stata, version 12 (StataCorp, College Station, Tex, USA). Analysis of questionnaire data will be descriptive and utilise univariate parametric and non-parametric analysis to identify differences between the control and intervention group and the intervention group prior to and following the intervention.

### Baseline characteristics

All known potential confounders will be measured at baseline, including sex, age, education level, marital status, and smoking status of partner and other household members. Thus, comparisons of the intervention and control groups will be performed both unadjusted and adjusted for these known confounders. This second adjusted analysis will control for any maldistribution after randomisation of the confounders between the two groups.

### Treatment effects

Analysis of the primary outcome will involve comparing the quit rate between the two groups. Simple unadjusted rates and 95% confidence intervals will be obtained in the first instance, with subsequent multiple regression analysis adjusting for other variables. Two forms of regression analysis will be considered for the primary outcome - logistic and Cox proportional hazard. Analysis of secondary outcomes will be conducted using standard statistical procedures applicable to categorical or continuous data. All tests of significance will be two-tailed.

### Procedures to account for missing data

Analysis will be on an intention-to-treat basis. A sensitivity analysis will be conducted for all participants who are lost to follow-up, with the assumption that none of them quit compared to all of them quitting at 12 months. An additional analysis will assume that participants in the intervention group who cannot be followed up will have the same quit rate as the control group. Participants who die during the trial will be excluded from the analysis.

### Ethics

The trial will be conducted in compliance with the protocol, the principles of Good Clinical Practice, the NHMRC National Statement on Ethical Conduct in Human Research (2007), NHMRC Australian Code for the Responsible Conduct of Research (2007), and NHMRC Values And Ethics: Guidelines For Ethical Conduct In Aboriginal And Torres Strait Islander Health Research (2003) [29-31]. This trial has ethical approval from The University of Western Australia HREC and the Western Australian Aboriginal Health Information and Ethics Committee, and support from the Kimberley Aboriginal Health Planning Forum Kimberley Research Subcommittee.

### Trial management

The Project Manager is responsible for the day to day management of the trial. The Project Manager's responsibilities include meeting regularly with the Aboriginal researchers and investigators, keeping copies of all documentation including details of standard care at DAHS and OVAHS and any changes to the standard Kimberley Smoking Cessation Protocol, making sure that the data is collected and stored properly, overseeing the recruitment of participants to the study, ensuring the randomisation and allocation process is complying with the protocol, recruit participants, administer the questionnaires and carry out follow ups if required, ensure project targets for participant enrolment and follow up are being met, and provide administrative support for the project.

The Aboriginal researchers will recruit participants, administer the questionnaires, carry out follow ups, keep copies of the questionnaires in a locked filing cabinet, and organise weekly case conferences with a clinic general physician and review all active participants in the intervention group.

## Protocol deviations

### Modification of the participant selection criteria and removing the Stages of Change model from the Protocol

A major selection criteria for this trial was that potential participants were required to be at the ready stage of the Stages of Change Model (i.e. considering quitting smoking soon / within the next 30 days). This model hypothesises that the process of someone quitting smoking is cyclical with smokers passing through the stages of NOT READY, UNSURE, READY, and quitting (MAINTENANCE), rather than a discreet event [32-34]. In this model smokers are thought to cycle through the stages of being ready, quitting and relapsing 3 to 4 times before successfully quitting long-term. The proponents of this model also suggest that interventions should be designed according to the stage the participants are at [33].

We found that this criterion is too restrictive and that very few Aboriginal peoples and Torres Strait Islanders in Derby and Kununurra fulfil the criteria for being at the 'READY' to quit smoking stage as we operationalised it, although a much larger number are interested in quitting. As detailed in the background, studies suggest that quitting can occur in what appear to be relatively unmotivated smokers and can be encouraged by intervention and the use of pharmacotherapy (reviewed in [16]). We therefore applied to the DSMC and supervising HREC to modify the selection criterion to include a wider range of people who are thinking of cutting down or quitting, so that we would be more likely to meet our sample size and to access a broader more appropriate target population.

## Discussion

Aboriginal peoples and Torres Strait Islanders smoke at much higher rates than non-Indigenous people and smoking is an important contributor to increased disease, hospital admissions and deaths in Indigenous Australian populations. Quit smoking programs in Australia have not had the same impact on Indigenous Australian smokers as on non-Indigenous smokers. A range of strategies have been used to encourage Indigenous people to quit smoking however there have been few good quality studies that show what approaches work best. More evidence of strategies that could work more widely in Indigenous Australian primary health care settings is needed if good policy is to be developed and implemented. This culturally appropriate, multidimensional, intensive smoking cessation intervention, which is provided by trained Aboriginal researchers in a remote Australian setting, has been designed to provide high quality evidence as to its efficacy. Part of the process of implementing high quality research pertinent to the health care needs of Aboriginal peoples and Torres Strait Islanders and to supporting a culture of translating such research into sustainable health care delivery requires support from the communities and health services involved. The involvement of Aboriginal community-controlled health services in projects such as this ensures local ownership, facilitates implementation, aids knowledge translation and builds local research capacity. The addition of a process evaluation of this study will provide important information about the type of research models that can work within ACCHS.

## Abbreviations

ACCHS: Aboriginal community controlled health service; BOABS: Be our ally, beat smoking; DAHS: Derby aboriginal health service; DSMC: Data safety and monitoring committee; HREC: Human research ethics committee; NRT: Nicotine replacement therapy, available in a range of forms such as patch, gum lozenges, tablets and nasal spray; NHMRC: National health and medical research council; OVAHS: Ord valley aboriginal health service; RCT: Randomised controlled trials.

## Competing interests

The authors declare that they have no competing interests.

## Authors' contributions

JM is an investigator. She contributed to the study design and drafted the first version of the study protocol, contributed to the development of the study questionnaires and the intervention, and to the study set-up. DA is an investigator. He contributed to the study design and protocol development. CN is an investigator. She contributed to the study design and protocol development. TK is an Aboriginal researcher. She contributed to the development of the locally-tailored and culturally appropriate smoking cessation program, the final study protocol and the questionnaires. DG is an investigator. He contributed to the design of the project. SM is an investigator. She contributed to the study design and protocol development. RM is an investigator. He contributed to the study design. GM is an investigator. He contributed to the study design and protocol development. All authors have provided critical review of this manuscript and have approved the final protocol.

## Pre-publication history

The pre-publication history for this paper can be accessed here:

http://www.biomedcentral.com/1471-2458/12/232/prepub

## References

[B1] Brady M Historical and cultural roots of tobacco use among Aboriginal and Torres Strait Islander people Aust N Z J Public Health 2002 26 2 120 124 12054329 10.1111/j.1467-842x.2002.tb00903.x

[B2] Ivers RG An evidence-based approach to planning tobacco interventions for Aboriginal people Drug Alcohol Rev 2004 23 1 5 9 10.1080/09595230410001645501 14965882

[B3] Australian Institute of Health and Welfare 2007 National Drug Strategy Household Survey: Detailed Findings Drug Statistics Series no 22 2008 Canberra: Australian Institute of Health and Welfare

[B4] Australian Bureau of Statistics National Aboriginal and Torres Strait Islander Health Survey, Australia, 2004-05 2006 Canberra: ABS

[B5] Gray D Stearne A Wilson M Doyle MF Indigenous-specific Alcohol and Other Drug Interventions: Continuities, Changes and Areas of Greatest Need 2010 Canberra: Australian National Council on Drugs

[B6] Gray D Morfitt B Ryan K Williams S The use of tobacco, alcohol and other drugs by young Aboriginal people in Albany, Western Australia Aust N Z J Public Health 1997 21 1 71 76 10.1111/j.1467-842X.1997.tb01657.x 9141733

[B7] Zubrick SR Silburn SR Lawrence DM Mitrou FG Dalby RB Blair EM Griffin J Milroy H De Maio JA Cox A The Western Australian Aboriginal Child Health Survey: The Social and Emotional Wellbeing of Aboriginal Children and Young People 2005 Perth: Curtin University of Technology and Telethon Institute for Child Health Research

[B8] Hymowitz N Cummings KM Hyland A Lynn WR Pechacek TF Hartwell TD Predictors of smoking cessation in a cohort of adult smokers followed for five years Tob Control 1997 6 Suppl 2 S57 S62 9583654 10.1136/tc.6.suppl_2.s57 PMC1766209

[B9] Unwin E Codde J Bartu A The impact of tobacco smoking on the health of Western Australians 2003 Perth: Health Department of Western Australia

[B10] American Diabetes Association Standards of medical care in diabetes--2006 Diabetes Care 2006 29 Suppl 1 S4 S42 16373931

[B11] Trewin D Madden R The Health and Welfare of Australia's Aboriginal and Torres Strait Islander Peoples 2005 Australian Bureau of Statistics & Australian Institute of Health and Welfare

[B12] Unwin E Thomson N Gracey M The impact of tobacco smoking and alcohol consumption on Aboriginal mortality and hospitalisation in Western Australia: 1983-1991 1994 Perth: Health Department of Western Australia 7746204

[B13] Lake P Aboriginal Attitudes to Smoking Aborig Isl Health Work J 1992 6 6 11 13

[B14] Pisinger C Vestbo J Borch-Johnsen K Jorgensen T Smoking cessation intervention in a large randomised population-based study. The Inter99 study Prev Med 2005 40 3 285 292 10.1016/j.ypmed.2004.06.001 15533541

[B15] Pisinger C Vestbo J Borch-Johnsen K Jorgensen T It is possible to help smokers in early motivational stages to quit. The Inter99 study Prev Med 2005 40 3 278 284 15533540 10.1016/j.ypmed.2004.06.011

[B16] Fagerstrom KO Can reduced smoking be a way for smokers not interested in quitting to actually quit? Respiration 2005 72 2 216 220 10.1159/000084057 15824536

[B17] DiGiacomo M Davidson PM Davison J Moore L Abbott P Stressful life events, resources, and access: key considerations in quitting smoking at an Aboriginal Medical Service Aust N Z J Public Health 2007 31 2 174 176 10.1111/j.1753-6405.2007.00037.x 17461010

[B18] Thomas D Johnston V Fitz J Lessons for Aboriginal tobacco control in remote communities: an evaluation of the Northern Territory 'Tobacco Project' Aust N Z J Public Health 2010 34 1 45 49 10.1111/j.1753-6405.2010.00472.x 20920104

[B19] Panaretto KS Lee HM Mitchell MR Larkins SL Manessis V Buettner PG Watson D Impact of a collaborative shared antenatal care program for urban Indigenous women: a prospective cohort study Med J Aust 2005 182 10 514 519 15896179 10.5694/j.1326-5377.2005.tb00017.x

[B20] Vos T Barker B Begg S Stanley L Lopez AD Burden of disease and injury in Aboriginal and Torres Strait Islander Peoples: the Indigenous health gap Int J Epidemiol 2009 38 2 470 477 19047078 10.1093/ije/dyn240

[B21] Couzos S Lea T Mueller R Murray R Culbong M Effectiveness of ototopical antibiotics for chronic suppurative otitis media in Aboriginal children: a community-based, multicentre, double-blind randomised controlled trial Med J Aust 2003 179 4 185 190 12914507 10.5694/j.1326-5377.2003.tb05496.x

[B22] Couzos S Lea T Murray R Culbong M 'We are not just participants-we are in charge': the NACCHO ear trial and the process for Aboriginal community-controlled health research Ethn Health 2005 10 2 91 111 10.1080/13557850500071038 15804658

[B23] Census of Population and Housing http://www.abs.gov.au/websitedbs/censushome.nsf/home/Census

[B24] Biddle N Ranking Regions: Revisiting an Index of Relative Indigenous Socioeconomic Outcomes 2009 Canberra: Centre for Aboriginal Economic Policy and Research, The Australian National University

[B25] Taylor J The Relative Socioeconomic Status of Indigenous People in the Kimberley. A Report to the Kimberley Land Council 2009 Canberra: Centre for Aboriginal Economic Policy and Research, The Australian National University

[B26] McDonald SP Maguire GP Hoy WE Validation of self-reported cigarette smoking in a remote Australian Aboriginal community Aust N Z J Public Health 2003 27 1 57 60 10.1111/j.1467-842X.2003.tb00380.x 14705268

[B27] Linacre S National Aboriginal and Torres Strait Islander Social Survey 2002 2004 Canberra: Australian Bureau of Statistics

[B28] NHMRC Australian Health Ethics Committee NHMRC Australian Health Ethics Committee (AHEC) Position Statement: Monitoring and reporting of safety for clinical trials involving therapeutic products 2009 Canberra: National Health and Medical Reserach Council

[B29] Therapeutic Goods Administration Note for Guidance on Good Clinical Practice (CPMP/ICH/135/95 - Annotaed with TGA comments) 2000

[B30] National Health and Medical Research Council Australian Research Council Australian Vice-Chancellors' Committee The National Statement on Ethical Conduct in Human Research 2007 Canberra: Australian Government

[B31] National Health and Medical Research Council Values and Ethics - Guidelines for Ethical Conduct in Aboriginal and Torres Strait Islander Health Research 2003 Canberra: Commonwealth of Australia

[B32] Prochaska JO DiClemente CC Velicer WF Ginpil S Norcross JC Predicting change in smoking status for self-changers Addict Behav 1985 10 4 395 406 10.1016/0306-4603(85)90036-X 4091072

[B33] Prochaska JO Goldstein MG Process of smoking cessation. Implications for clinicians Clin Chest Med 1991 12 4 727 735 1747990

[B34] Prochaska JO Velicer WF The transtheoretical model of health behavior change Am J Health Promot 1997 12 1 38 48 10.4278/0890-1171-12.1.38 10170434

